# Hidden Carbenes: NHC-Al Species at the Al_2_O_3_–Ionic Liquid Interface

**DOI:** 10.1021/jacsau.5c00736

**Published:** 2025-07-26

**Authors:** Muhammad I. Qadir, Camila P. Ebersol, Blendo A. da Silva, Marcus V. Castegnaro, Luciano M. Lião, Flávio O. Sanches-Neto, Heibbe Cristhian B. de Oliveira, Renato B. Pontes, Gunter Ebeling, Jairton Dupont

**Affiliations:** † Instituto de Quimica-Universidade Federal de Goias-UFG, Avenue Esperanca s/n, Câmpus Samambaia, Goiânia, Goias 74690-900, Brazil; ‡ Institute of Physic, 28124 Universidade Federal do Rio Grande do Sul (UFRGS), Avenue Bento Gonçalves, 9500, Porto Alegre, Rio Grande do Sul 91501-970, Brazil; § Instituto de Fisica-Universidade Federal de Goias-UFG, Avenue, Esperança s/n, Campus Samambaia, Goiânia, Goias 74690-900, Brazil; ∥ Institute of Chemistry-Universidade Federal do Rio Grande do Sul-UFRGS, Avenue Bento Gonçalves, 9500 Porto Alegre, Porto Alegre, Rio Grande do Sul 91501-970, Brazil; ⊥ Departamento de Bioquimica y Biologia Molecular B e Inmunologia Facultad de Quimica, Universidad de Murcia, P.O. Box 4021, Murcia E-30100, Spain

**Keywords:** aluminum N-heterocyclic carbenes, ionic liquids geometry, SILPs, electronic modifications, solid-state NMRs

## Abstract

The modification of the surface chemistry of heterogeneous catalysts/supports alters their electronic and catalytic properties, particularly through the incorporation of a monolayer of sophisticated N-heterocyclic carbenes (NHCs). However, the formation of aluminum N-heterocyclic carbene (NHC-Al) goes unrecognized or remains unidentified when imidazolium-based ionic liquids are supported on solids (SILPs). In this work, we identified the formation of NHC-Al species upon the grafting of imidazolium-based ILs onto neutral Al_2_O_3_. The optimal geometry of the imidazolium cations in these SILPs adopts a tilted orientation, exhibiting an upright binding mode, akin to self-assembled monolayers (SAMs). This configuration enables direct interaction of the electron-rich N–C–N moiety of the imidazolium cation with the Al_2_O_3_ surface, leading to the autocatalytic formation of NHC-Al species. The NHC-Al species are confirmed through solid-state ^13^C NMR, ^13^C–^1^H HETCOR, ^27^Al NMR, synchrotron XPS, XANES, and DFT calculations. This study provides unique insights into the bonding and structural geometry of ILs in SILPs, revealing features that have previously gone unobserved.

## Graphical abstract

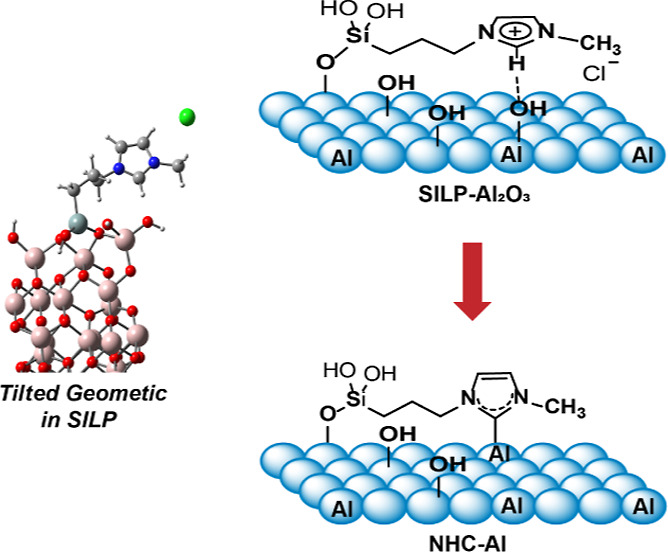

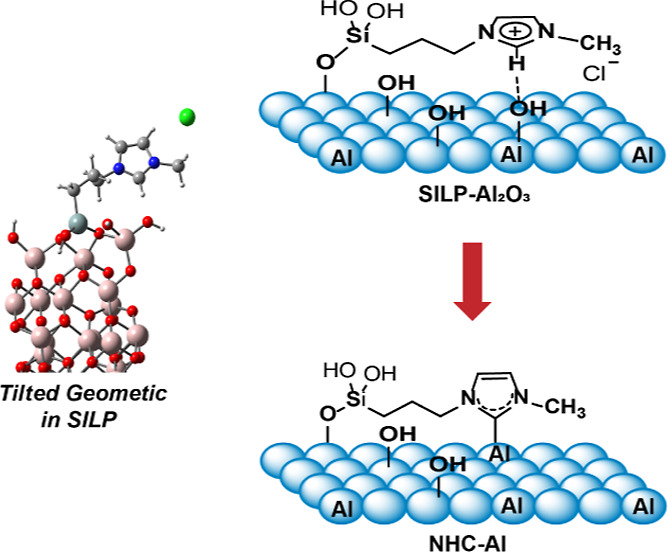

## 1 Introduction

Supported ionic liquid phased (SILPs) and solid catalysts with IL-layer (SCILL) are hybrid organic–inorganic devices that contain a homogeneous film of ionic liquids onto the solids (SiO_2_ and Al_2_O_3_, for instance).^1–4^ A monolayer of IL in SILPs forms a cage or membrane around the guest complex or metal nanoparticle, acting as a catalytically active membrane that controls the diffusion of reactants, intermediates, and products to the catalytic active sites.^5–8^ However, very little is known about the morphology and phase behavior of ILs when they interact with solid supports. Surface polarization of solid supports (SiO_2_) can be induced through the formation of hydrogen bonds between the hydrogen-bond networks of ILs and surface hydroxyl groups.^9^ Imidazolium-based IL ions are generally asymmetric and flexible, with delocalized electrostatic charges that can enhance the surface local density and tune the hydrophobicity or hydrophilicity of the IL near the support surface, facilitating substantial charge transfer.^10,11^

N-heterocyclic carbenes (NHC) have been broadly used as ligands in metal complexion as well as capping and stabilizing agents for metal nanoparticles.^12^ They are electron-rich and function as nucleophiles with efficient electron-donating characteristics, particularly benzimidazolium and imidazolium types. The cationic charge is delocalized within the five-membered imidazolium rings, which enhances the electron-donating character of the nitrogen atoms.^13^ Few studies have investigated the self-assembly of N-heterocyclic imines on planar metal surfaces such as gold, silver, palladium, copper, and silicon.^14–22^

Although the electron-donating behavior of NHCs and their binding modes (both flat-type and upright) have been examined on metal surfaces, the binding modes and interfacial charge transfer of imidazolium-based ionic liquids (ILs) have not yet been explored. In fact, NHCs attach to CuO_*x*_ via coordination with surface oxygen atoms, while their interaction with TiO_*x*_ occurs through coordination with surface metal atoms.^23^ In contrast, NHCs bind to FeO_*x*_ by coordinating with both surface metal and oxygen atoms.^23^ These distinct binding modes of the NHCs are attributed to variations in the electronic properties of the metal atoms in the metal-oxide films studied.

In this work, we represent the binding geometries of the imidazolium- and pyridinium-based IL on the Al_2_O_3_ surface (Scheme 1b). Simple covalently bonded imidazolium- and pyridinium-based SILPs of Al_2_O_3_ were prepared. The imidazolium cation adopts an upright adsorption geometry, exhibiting high electron and charge density on the Al_2_O_3_ surface, which leads to the formation of aluminum N-heterocyclic carbene (NHC-Al), alongside neutral SILPs (NHC@SILP). The formation of NHC-Al species in NHC@SILP-PMImAl_2_O_3_ is deeply investigated through solid-state ^13^C NMR, ^13^C–^1^H HETCOR, ^27^Al NMR, and synchrotron depth-profiling XPS and XANES measurements.

**Scheme 1:**
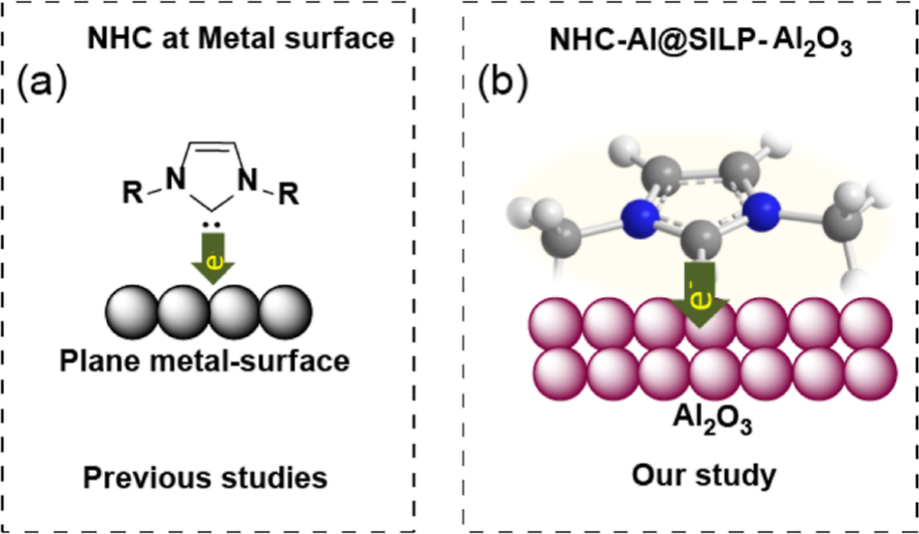
(a) Binding Mode of NHC on the Metal Surface (Previous Work)^14–18,24,25^ and (b) Bind Mode of Our Imidazolium SILP-Al_2_O_3_ (This Work)

## 2 Results and Discussion

Al_2_O_3_-supported SILPs were prepared using a sol–gel process by the reaction of 1-methyl-3-(3-trimethoxysilylpropyl)-imidazolium chloride (MPrSiMIm.Cl) and *N*-(3-trimethoxysilylpropyl)-pyridinium chloride ILs with neutral Al_2_O_3_, following modifications to previous methods.^26^ Solid-state ^13^C and ^29^Si CP-MAS analyses of the NHC@SILP-PMImAl_2_O_3_ and SILP-PPyAl_2_O_3_ catalysts confirmed that the ILs were covalently bonded to the Al_2_O_3_ (Figures S1–S3, see the Supporting Information).

SEM images showed that Al_2_O_3_ is composed of irregularly shaped particles, and the EDS chemical mapping indicated the presence of Al, C, N, Cl, O, and Si, which showed that the ILs were homogeneously anchored on the surface of Al_2_O_3_ (Figures S4–S5). BET analysis reveals a decrease in surface area and pore volume from 109.7 m^2^/g for pristine Al_2_O_3_ to 16.3 and 15.2 m^2^/g for the NHC@SILP-PMImAl_2_O_3_ and SILP-PPyAl_2_O_3_ catalysts, respectively (Table S1, Figure S6). This reduction is attributed to the incorporation of the ionic liquid, which likely leads to micropore filling and/or blockage.

To identify the energetically preferred binding geometry of the contact-ion pair of ILs in our SILPs, DFT simulations were performed. The optimal geometry of the imidazolium cation in NHC@SILP-PMImAl_2_O_3_ was found to be in a tilted position, exhibiting an upright binding mode (Figure 1a,c). This tilted configuration allows the electron-rich N–C–N bond to come into direct contact with the surface. The bond distance between the 2C–H bond of the imidazolium cation and the oxygen bond of the surface hydroxyl group of Al_2_O_3_ was 1.80 Å. A similar tilted geometry was also observed for the pyridinium-based IL (SILP-PPyAl_2_O_3_), with the pyridinium cation adopting an upright geometry but positioned slightly away from the surface (Figure 1b,d). A bond distance of 1.86 Å was found between the aromatic C–H bond of the electron-deficient pyridinium ring and the oxygen group of the –OH group on Al_2_O_3_. The NHCs also exhibited an upright geometry when directly bound to the metal surfaces of Au, Ag, and Si through their strong σ-bonds as stabilizing ligands.^17,27,28^

**Figure 1:**
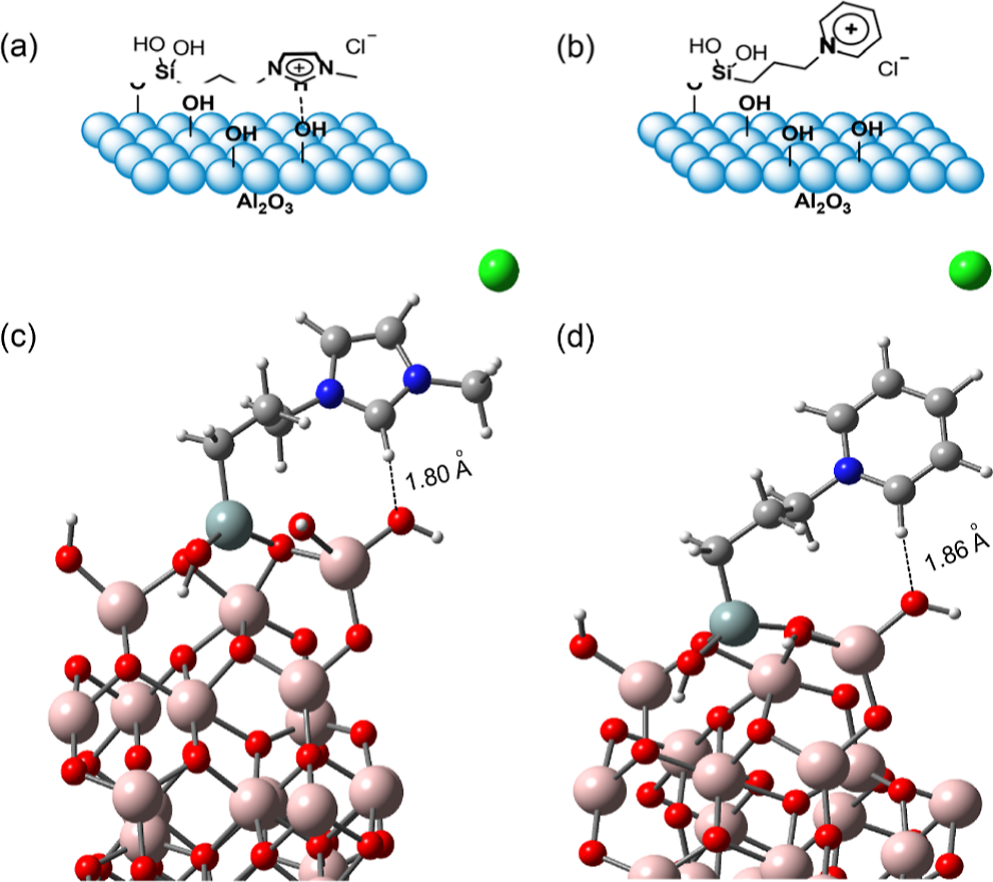
(a) Schematic representation of the binding geometry and (c) DFT-optimized geometry of the imidazolium cation in imidazolium SILP-PMImAl_2_O_3_. (b) Schematic representation of the binding geometry and (d) DFT-optimized geometry of the pyridinium cation in SILP-PPyAl_2_O_3_. (Considering IL SILP-PMImAl_2_O_3_.)

It was observed that the tilted configuration caused coordination of the imidazolium cation of SILP-PMImAl_2_O_3_ to aluminum and generated the NHC-Al adduct (NHC@SILP-PMImAl_2_O_3_). Solid-state ^13^C CP-MAS NMR analysis showed a peak at 171.2 ppm attributed to the imidazolium carbene (Figure 2a), which is coordinated to aluminum.^29^ The signals of the methylimidazolium cation can be seen at 43.7, 121.9, and 135.7 ppm, while peaks at δ = 17.6, 31.9, and 59.2 ppm belong to the propylene moiety.

**Figure 2:**
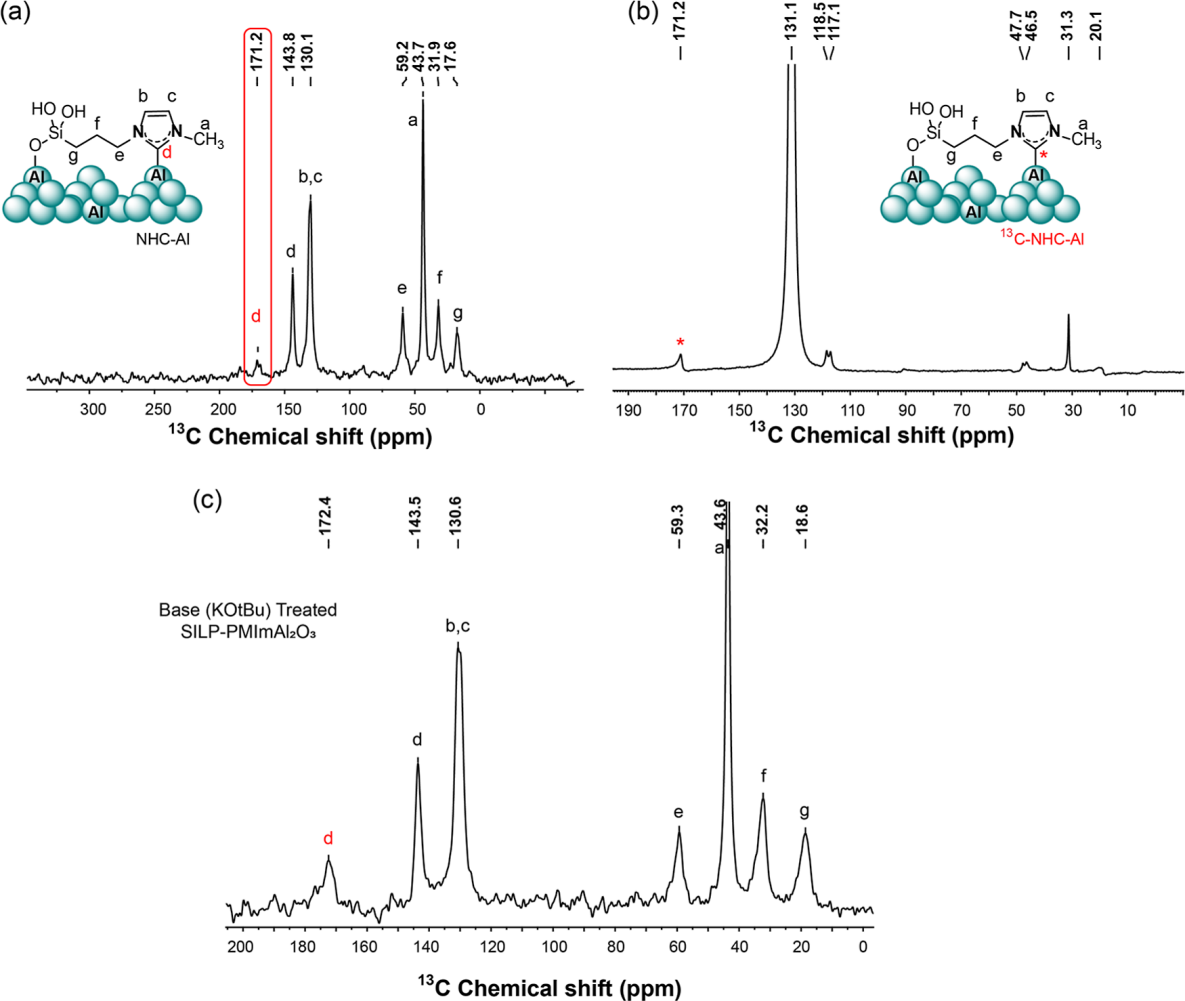
(a) High-resolution solid-state ^13^C CP-MAS NMR spectra of NHC@SILP-PMImAl_2_O_3_, (b) solid-state ^13^C CP-MAS NMR spectra of ^13^C-enriched NHC@SILP-PMImAl_2_O_3_, and (c) ^13^C CP-MAS NMR spectra of SILP-PMImAl_2_O_3_ after treatment with KOtBu base.

This represented the presence of a mixture of carbene imidazolium adduct and IL, giving the name NHC@SILP-PMImAl_2_O_3_ to SILP-PMImAl_2_O_3_. Furthermore, a ^13^C2-enriched NHC@SILP-PMImAl_2_O_3_ featuring a ^13^C-enriched H–C2 position on the imidazolium cation, was prepared, which showed an intense peak at 171.2 ppm, attributed to the carbene carbon, was observed (Figure 2b). Of note, the ^13^C NMR spectrum of the simply incorporated ^13^C-enriched *n*-butyl-3-methylimidazolium chloride (labeled at the H–C2 position) on Al_2_O_3_ did not show the formation of NHC-Al species (Figure S7). To compare our generated NHC-Al, we treated SILP-PMImAl_2_O_3_ with KOtBu, a strong base, resulting in the formation of NHC-Al. The solid-state ^13^C CP-MAS NMR spectrum (Figure 2c) clearly shows an intense signal at approximately 172 ppm, which is characteristic of NHC-Al.

The 2D ^1^H–^13^C HETCOR experiments provide good resolution of the correlation between carbon and its bonded hydrogen, as shown in Figure S8. The carbon resonance signals present in the imidazole and carbon chain, along with their associated protons, are in good agreement. Notably, the absence of a carbon peak at around 172 ppm affirms the presence of the carbene adduct.

Figure 3a shows the high-resolution MAS ^27^Al NMR analysis of NHC@SILP-PMImAl_2_O_3_ exhibiting two characteristic features of Al_2_O_3_ at 10.0 and 73.5 ppm, representing the presence of Al^3+^ ions in octahedral and tetrahedral coordination, respectively.^30,31^ A prominent peak was observed at 123.9 ppm, corresponding to the NHC-Al adduct.^32^

**Figure 3:**
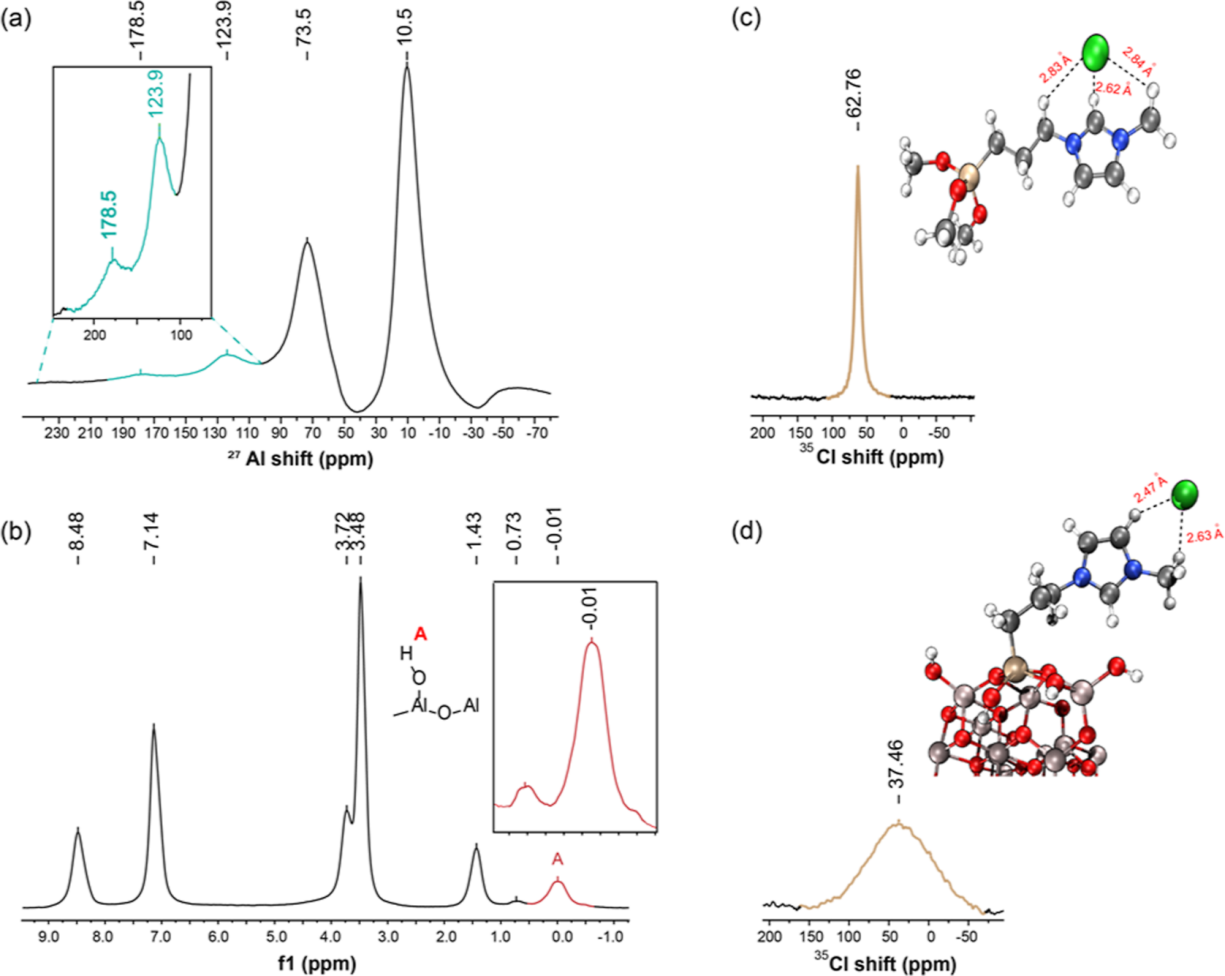
High-resolution (a) solid-state ^27^Al MAS NMR of NHC@SILP-PMImAl_2_O_3_, (b) solid-state ^1^H MAS NMR of NHC@SILP-PMImAl_2_O_3_, (c) liquid ^35^Cl NMR with DFT-optimized geometry of the imidazolium cation in MPrSiMIm.Cl ionic liquid, and (d) solid-state ^35^Cl MAS NMR spectra with DFT-optimized geometry of the imidazolium cation in NHC@SILP-PMImAl_2_O_3_.

The ^35^Cl NMR analysis was performed that showed a broad peak at around 37 ppm for NHC@SILP-PMImAl_2_O_3_ while a sharp peak at 62.0 ppm was found for 1-methyl-3-(3-trimethoxysilylpropyl)-imidazolium chloride bulk IL (Figure 3c,d). The Cl^–^ counterion likely experiences a more complex environment, including potential interactions with surface Al species, which can contribute to the broadening observed in the ^35^Cl NMR. In addition to these interactions, other factors such as H-bonding and hydrophilicity of the IL may also play a role. The difference in sharpness or broadness may be related to hydrogen bonding and the Cl–H distance in the contact-ion pair of the IL, as observed in the DFT-simulated Figure 3c,d. In bulk IL, Cl is in H-bonding with three carbons, C2–H, –CH_3_, and –CH_2_, which represents the closely packed-contact-ion pair, while in NHC@SILP-PMImAl_2_O_3_, there are just two H-bondings with Cl (H–C2 and –CH_3_), suggesting a less compact ion pair environment.

Synchrotron AR-XPS analysis was performed to determine the chemical environment of the surface atoms in SILPs.^33–35^ Figure 4a shows the XPS spectra for the C 1s and N 1s deconvolutions. The C 1s spectra represent the peaks of the carbon skeleton, which are in different chemical environments of the imidazolium^36^ and pyridinium cations.^37^ A high-intensity peak at 285.0 eV corresponds to the aliphatic carbons. The peak at 286.8 eV is ascribed to the C–N moieties, while the peak around 287.5 eV originates from the C-2 of the imidazolium cation.^38^

**Figure 4:**
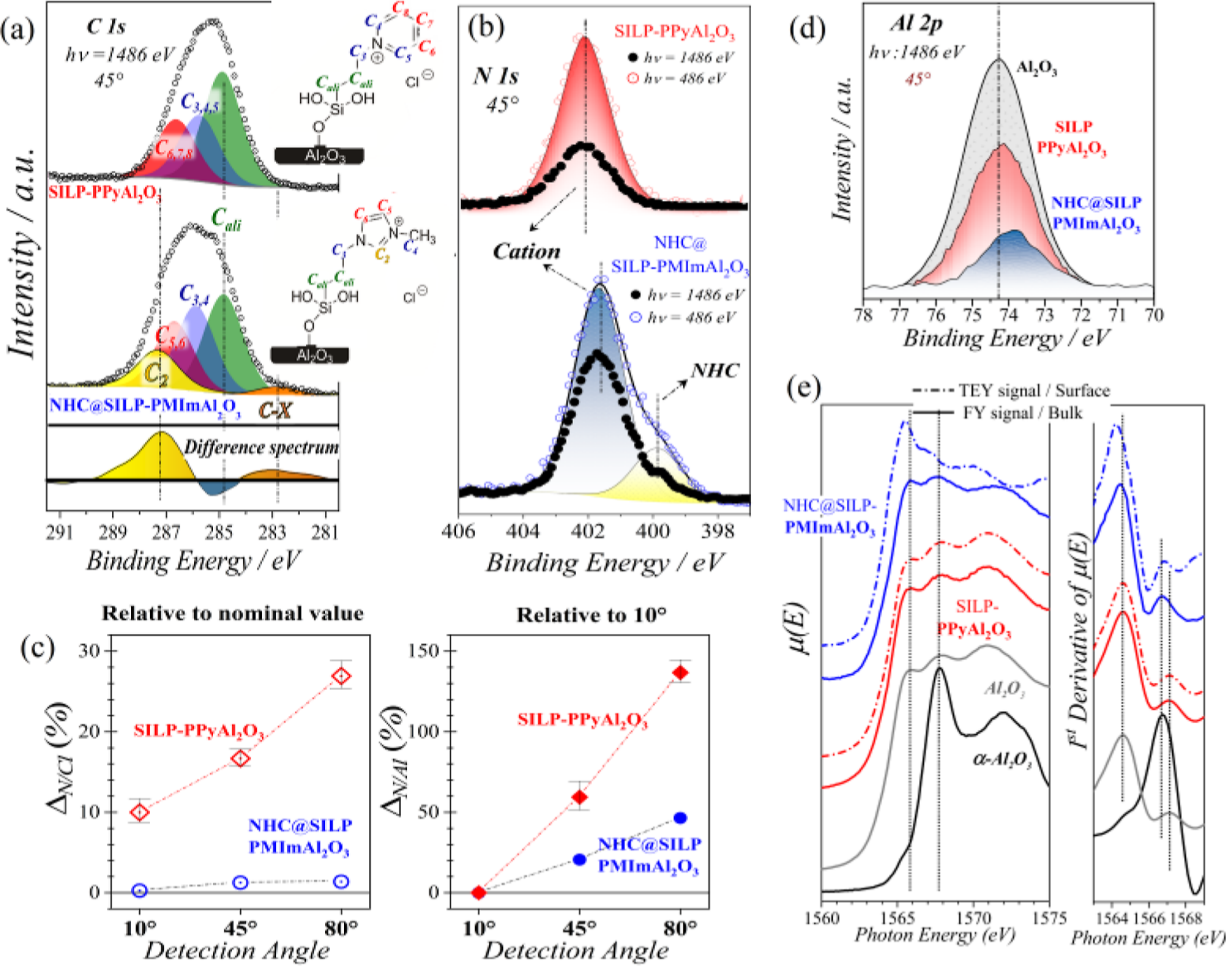
Synchrotron XPS analysis of NHC@SILP-PMImAl_2_O_3_ and SILP-PMImAl_2_O_3_, (a) C 1s region, (b) N 1s region, (c) Al 2p_3/2_ region, and (d) comparison of the N/Al and Cl/Al depth distribution of NHC@SILP-PMImAl_2_O_3_ and SILP-PMImAl_2_O_3_. (e) Comparison of the Al K-edge XANES spectra of NHC@SILP-PMImAl_2_O_3_ and SILP-PMImAl_2_O_3_.

N 1s XPS spectra were collected using two different excitation energies (1486 and 486 eV) to tune the surface sensitivity by selecting the kinetic energies of the photoelectrons emitted from the N core level (Figure 4b). The N 1s region displayed a single peak at a binding energy of 401.7 eV, which corresponds to the nitrogen atoms of the pyridinium and imidazolium ring.^37,38^ Notably, in the N 1s region, a prominent peak at 400.1 eV was observed, which is attributed to the NHC-Al structure and confirms its formation in our NHC@SILP-PMImAl_2_O_3_. A similar coordination/formation of NHCs with metal nanoparticles has been observed earlier.^39,40^ A small peak at 400.1 eV and a larger one at 401.7 eV indicate the presence of a mixture of NHC and IL in our imidazolium SILP. Increasing the surface sensitivity of the N 1s spectra, by reducing the excitation energy from 1486 to 486 eV, enhanced the contributions at a low binding energy. This indicates an enrichment of NHC-Al species at the outermost surface layers, highlighting stronger IL–substrate interactions in NHC@SILP-PMImAl_2_O_3_ compared to SILP-PPyAl_2_O_3_.

A more detailed understanding of the depth distribution of elements in NHC@SILP-PMImAl_2_O_3_ and SILP-PPyAl_2_O_3_ was achieved via AR-XPS collected at detection angles of 10°, 45°, and 80° (Figures 4d and S9–S12). The depth dependences of the N/Al ratio for each sample are compared in the bottom panel of Figure 4c by the relative variation with respect to the respective N/Al ratio values at 0°. For NHC@SILP-PMImAl_2_O_3_, distinct variations in N/Al and N/Cl ratios across the near-surface region supported the conclusion that the imidazolium-based SILP has more stable and uniform IL coverage compared to SILP-PPyAl_2_O_3_, attributed to strong interactions between the imidazolium cation and surface. This result is consistent with DFT calculations, which shows that the IL cation assumes a tilted geometrical orientation upon the Al_2_O_3_, not in a flagpole-like orientation.

The presence of NHC-Al species is also evident in the 0.5 eV downshift observed in the Al 2p_3/2_ region for NHC@SILP-PMImAl_2_O_3_ (Figure 4b), which is consistent with increased electron density at Al atoms due to NHC coordination, likely due to charge transfer from the contact-ion pair of the ionic liquid. This observation was further validated by XANES at the Al K-edge (Figure 4e).^41,42^ As expected, the SILP spectra closely resembled that of pristine Al_2_O_3_ collected in bulk-sensitive FY (fluorescence yield) mode, confirming unaltered bulk-Al coordination (mixed octahedral and tetrahedral sites). However, the surface-sensitive TEY (total electron yield) signal of NHC@SILP-PMImAl_2_O_3_ showed a 0.4 eV shift toward lower energies, corroborated by the first-derivative spectra, reflecting the increased electron density at Al sites. Furthermore, the distinct fine structures in the TEY signal of NHC@SILP-PMImAl_2_O_3_ indicate that NHC-Al interactions also increase the proportion of less-coordinated Al sites at the surface. The TEY-FY comparison confirms that these modifications are localized to the outermost surface, where the imidazolium influences the ligand field around the Al species. The combination of XANES and XPS revealed that the surface of imidazolium-modified Al_2_O_3_ presents lower-coordinated Al sites with a higher electron density due to NHC-Al formation.

### 2.1 A Plausible Mechanism of NHC-Al Formation

The reaction mechanism for the formation of NHC-Al proposed in NHC@SILP-PMImAl_2_O_3_ involves deprotonation and metalation steps (Figure 5).

**Figure 5:**
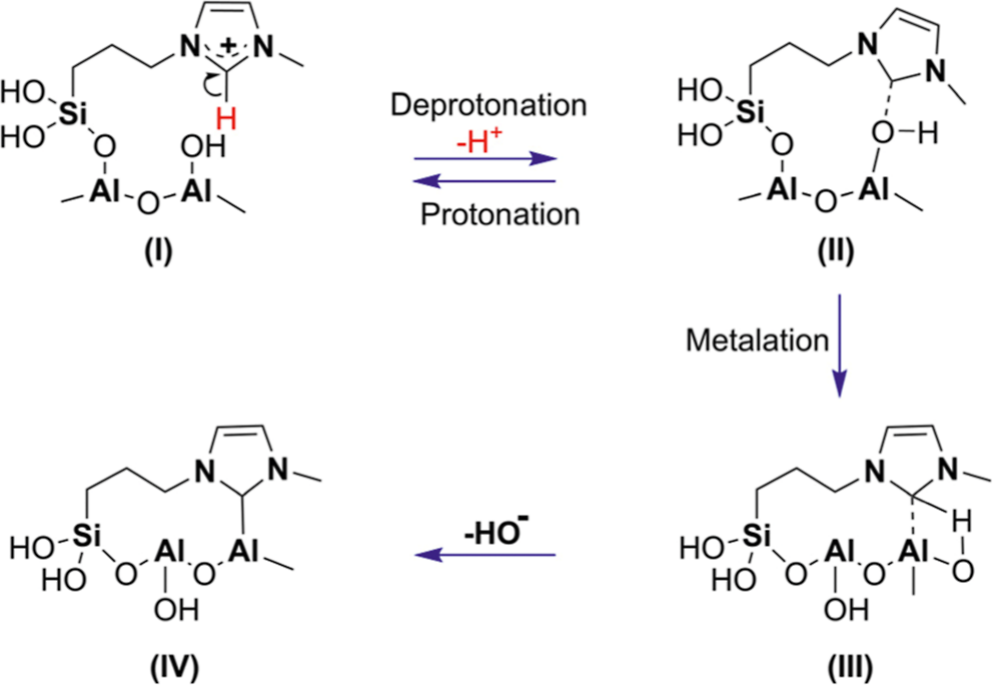
A simple proposed mechanism for the formation of N-heterocyclic carbene of aluminum (NHC-Al) in our NHC@SILP-PMImAl_2_O_3_.

The first step may involve thermally induced autocatalytic deprotonation,^43,44^ resulting in the generation of carbene (II), under our reaction conditions used for incorporating IL on Al_2_O_3_. The formed NHC coordinates with the surface OH group, as observed previously.^23^ The next step follows an exergonic metalation and generates NHC-Al (IV). A similar mechanistic approach has been observed when NHC is generated on Ag_2_O.^45,46^ On the other hand, water can be dissociated on the Al_2_O_3_ surface to generate stable hydroxides.^47–49^ Since SILPs contain confined/residual H_2_O molecules, which may undergo dissociation on Al_2_O_3_ and generate a hydroxides layer on Al_2_O_3_. These hydroxides can abstract the H–C2 proton and form a carbene adduct (II), which then undergoes metalation to generate NHC-Al (IV).

DFT calculations were performed to study the interaction/absorption of the 1-n-butyl-3-methylimidazolium carbene adduct (NHC) onto Al_2_O_3_. Figure 6 shows two representative structures of the adsorption of this carbene on the Al_2_O_3_ cluster. We consider two distinct conformations: (i) fully hydroxylated Al_2_O_3_ cluster (Figure 6a) and (ii) three OH groups were removed from the Al_2_O_3_ cluster (Figure 6b) aiming to maximize the interaction between the molecule and an Al atom of the surface. The calculated adsorptions energies were −1.65 and −1.96 eV, respectively. Thus, our findings clearly indicate that the 1-n-butyl-3-methylimidazolium carbene interacts slightly stronger with the Al_2_O_3_ when Al atoms are exposed, i.e., no OH groups in the vicinity.

**Figure 6:**
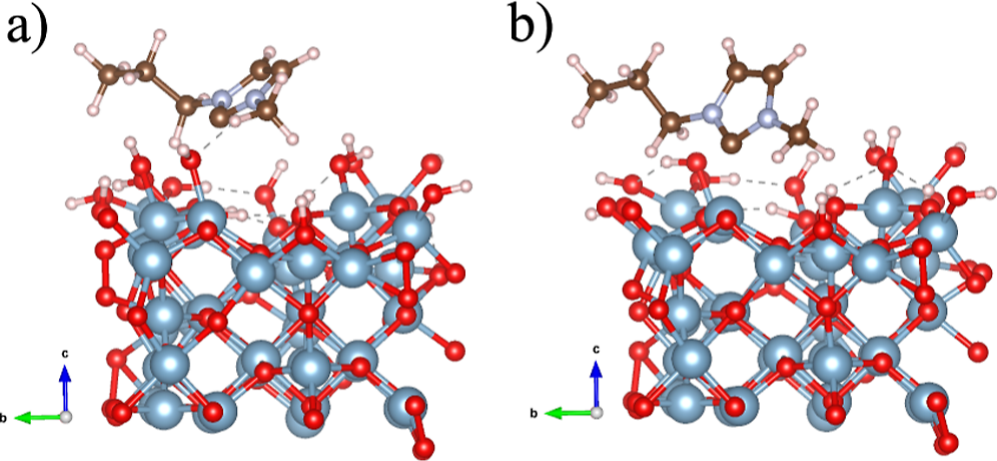
Adsorption energies of coordination modes of 1-n-butyl-3-methylimidazolium carbene (NHC) onto the Al_2_O_3_ cluster: (a) adsorption on the fully hydroxylated cluster and (b) three OH groups were removed from the cluster.

## 3 Conclusions

We have revealed the formation of aluminum N-heterocyclic carbene (NHC-Al) in a simple imidazolium-based SILP of Al_2_O_3_. The pyridinium and imidazolium are in a tilted position in their SILPs. This geometry allows the incorporation of the electron-rich N–C–N bond of the imidazolium cation directly to Al_2_O_3_ and results in the autocatalytic formation of N-heterocyclic carbene (NHC-Al), confirmed by solid-state NMRs, synchrotron XPS, XANES, and DFT calculations. This work provides insights into the IL bonding and structural geometry in SILPs, and these findings provide new insights into the structural organization and bonding environment of ILs in SILPs, unveiling molecular-level phenomena that have remained undetected by conventional methods. It is evident that N-heterocyclic carbenes (NHCs) can form even on “neutral” alumina surfaces, particularly when imidazolium moieties are grafted. This highlights the need for careful consideration of the basicity of the solvents used during SILP preparation as basic media may promote the generation of surface-bound NHC species.

## Supplementary Material



## Notes

Detail information on the preparation of SILPs, solid-state ^29^Si CP-MAS analysis of NHC@SILP-PMImAl_2_O_3_ and SILP-PPyAl_2_O_3_, SEM and chemical mapping of NHC@SILP-PMImAl_2_O_3_ and SILP-PPy-Al_2_O_3_ catalyst, BET analysis and DFT calculations, solid-state ^13^C-MAS NMR spectra of coated carbon-13-enriched BMIm.Cl on Al_2_O_3_, 2D ^1^H–^13^C HETCOR solid-state NMR spectra of NHC@SILP-PMImAl_2_O_3_, surface composition of SILPs by AR-XPS analysis, FTIR analysis of SILPs, and DFT calculations for adsorption energies of coordination modes of NHCs (PDF)
