# Supplementary material for: Hidden Carbenes: NHC-Al Species at the Al2O3–Ionic Liquid Interface
Source: JACS Au. 2025 Jul 26;5(9):4370–7. doi: 10.1021/jacsau.5c00736 (PMC12457999; doi:10.1021/jacsau.5c00736)
Supplement: Supplementary file 1 [file au5c00736_si_001.pdf]

Supporting Information

Hidden Carbenes: NHC-Al Species at the AL_2O_3-Ionic Liquid Interface

Muhammad I. Qadir,*,† Camila P. Ebersol,† Blendo A. da Silva,† Marcus V. Castegnaro,‡ Luciano M. Lião,† Flávio O. Sanches-Neto,† Heibbe Cristhian B. de Oliveira,† Renato B. Pontes,¶ Gunter Ebeling,§ Jairton Dupont *,§,#

† Instituto de Química-Universidade Federal de Goiás-UFG-Av. Esperança s/n, Câmpus Samambaia. 74690-900, Goiânia, Goiás, Brazil

‡ Institute of Physic, Universidade Federal do Rio Grande do Sul (UFRGS), Av. Bento Gonçalves, 9500, Porto Alegre 91501-970, RS, Brazil.

¶ Instituto de Fisica-Universidade Federal de Goiás-UFG-Av. Esperança s/n, Campus Samambaia, 74690-900, Goiânia, Goiás, Brazil.

§ Institute of Chemistry-Universidade Federal do Rio Grande do Sul-UFRGS-Av. Bento Gonçalves, 9500 Porto Alegre 91501-970, Porto Alegre, RS, Brazil

Departamento de Bioquímica y Biología Molecular B e Inmunología Facultad de Química, Universidad de Murcia, P.O. Box 4021, E-30100 Murcia, Spain

Contents **Page**

Contents	Page
1- Preparation of SILPs	2
2- Solid state ^{13}C and ^{29}Si CP-MAS analysis of SILP-PPyAl_2O_3	2
3- Solid state ^{29}Si CP-MAS analysis NHC@SILP-PMImAl_2O_3	4
4. SEM and Chemical mapping of NHC@SILP-PMImAl_2O_3 and SILP-PPy-Al_2O_3 catalyst	4
5. BET analysis of SILP-Al_2O_3	5
6. DFT calculations	5
7- Solid state ^{13}C-MAS NMR spectra of coated carbon-13 enriched BMIm.Cl on Al_2O_3.	6
8- 2D ^{1}H-^{13}C HETCOR solid state NMR spectra of NHC@SILP-PMImAl_2O_3	8
9. Surface composition of SILPs by AR-XPS analysis	9
10. FTIR analysis of SILPs	13
11. DFT calculations for Adsorption energies of coordination modes of NHCs	14
12. Reference	14

1- Preparation of SILPs

The SILP (NHC@SILP-PMImAl$_2$O$_3$) was prepared using reported method with modifications.[1] In first step, 1-methyl-3-(3-trimethoxysilylpropyl)-imidazolium chloride IL was prepared by mixing the 1-methylimidazol (10.0 mmol) and (3-Chloropropyl)trimethoxysilane (11.0 mmol) at 90 °C for 72 h. Then the mixture was washed with dried diethyl ether (3x20 mL). The solvent was removed under vacuum. In second step, 2.0 g of IL and 7.0 g of Al$_2$O$_3$ (neutral, 70-230 mesh) in CH$_3$CN (50 mL) was refluxed at 120 °C for 72 h. Afterward, the solid was isolated by filtration, washed it with acetone (3x20 ml) and dried at 60 °C for 2 h. The pyridinium based-SILP (SILP-PPyAl$_2$O$_3$) was also prepared by following the above methodology using pyridine (10.0 mmol) and 3-Chloropropyl)trimethoxysilane (11.0 mmol).

OBs: The Al$_2$O$_3$ was calcinated at 200 °C with 5 °C ramp/min for 2h before using for the incorporation of ILs.

Abs: Base treated SILP was prepared by missing 200 mg of SILP-PMImAl$_2$O$_3$ in solution of KOtBu (1 mml in 5 mL of methanol). The solid was filtered and dried at 80 °C for 2h.

2- Solid state ^{13}C and ^{29}Si CP-MAS analysis of SILP-PPyAl$_2$O$_3$

^{13}C CP-MAS of SILP-PPyAl$_2$O$_3$ catalyst demonstrated peaks at δ = 17.49, 34.45, 70.97 135.48 and 152.12 ppm attributed to the propylene moiety and pyridinium cation. While a peak at 53.20 ppm attributed to a one -OCH$_3$ group. ^{29}Si CP-MAS NMR spectrum of SILP-PPyAl$_2$O$_3$ showed peaks at δ = -66.22 and -68.15 ppm are attributed to the Si atoms, attached covalently Al$_2$O$_3$.

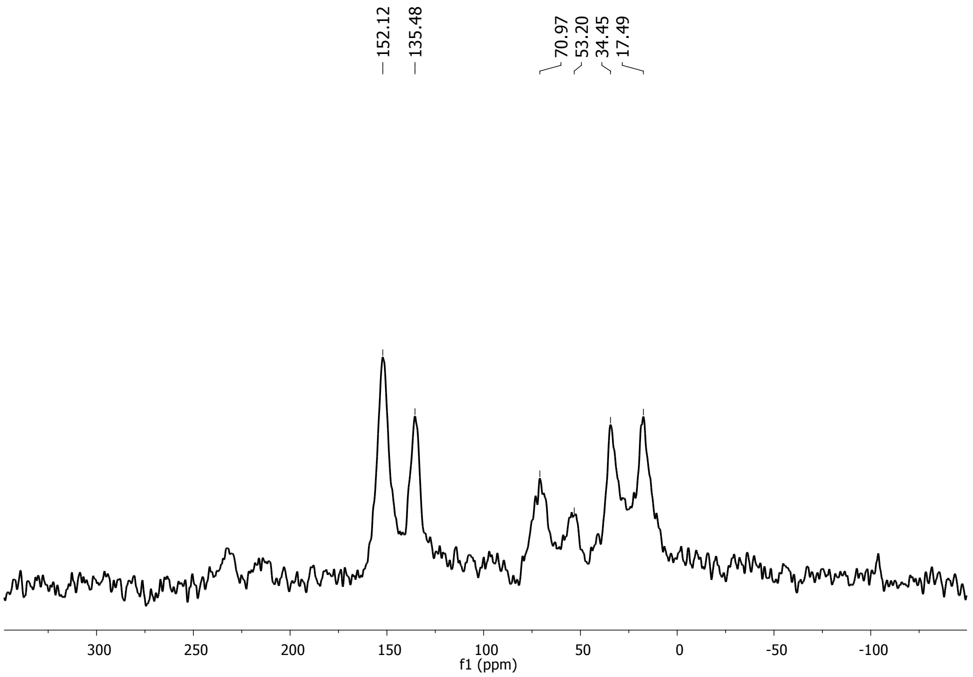

Figure S1. ^{13}C CP-MAS spectra of SILP-PPyAl$_2$O$_3$ catalyst

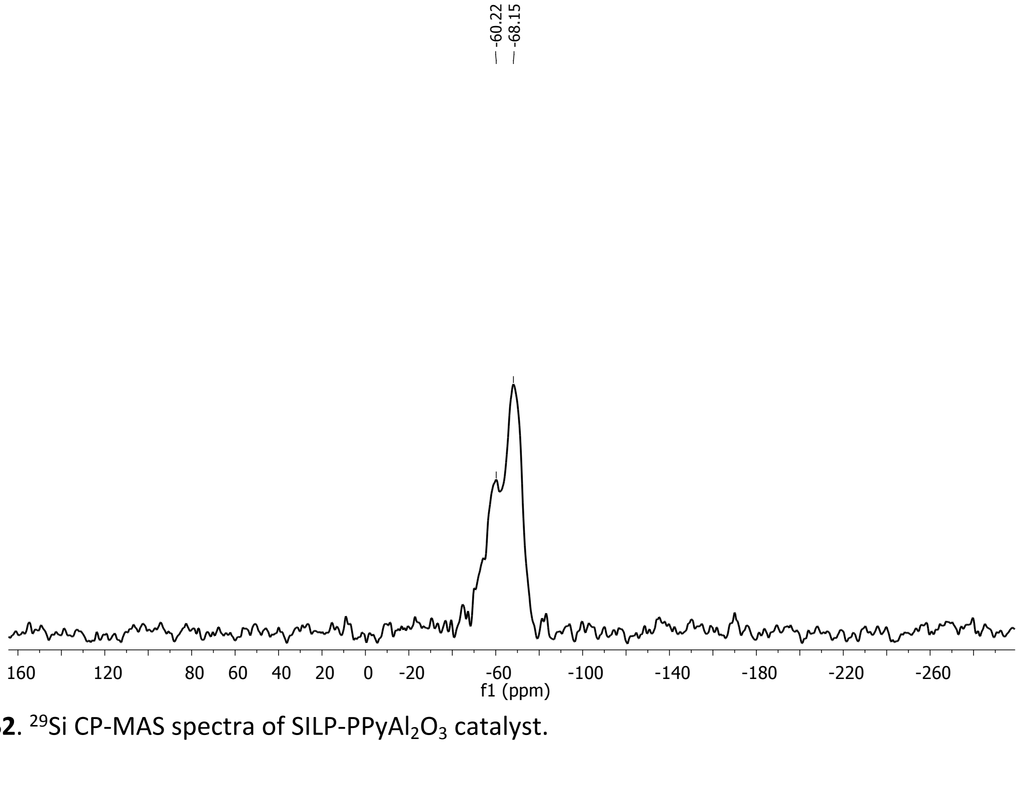

Figure S2. ^{29}Si CP-MAS spectra of SILP-PPyAl$_2$O$_3$ catalyst.

3- Solid state ^{29}Si CP-MAS analysis NHC@SILP-PMImAl$_2$O$_3$

^{29}Si CP-MAS NMR spectrum of SILP-PMImAl$_2$O$_3$ represented peaks at δ = -58.31 and -68.08 ppm are attributed to the Si atoms, attached covalently to Al$_2$O$_3$.

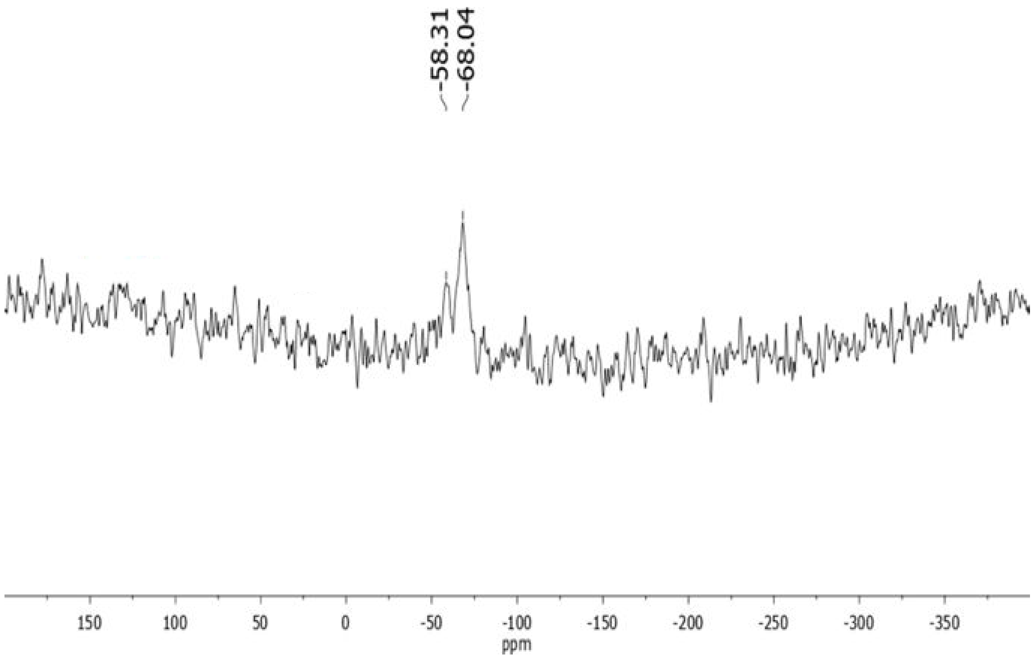

Figure S3. ^{29}Si CP-MAS spectra of NHC@SILP-PMImAl$_2$O$_3$ catalyst

4. SEM and Chemical mapping of NHC@SILP-PMImAl$_2$O$_3$ and SILP-PPy-Al$_2$O$_3$ catalyst

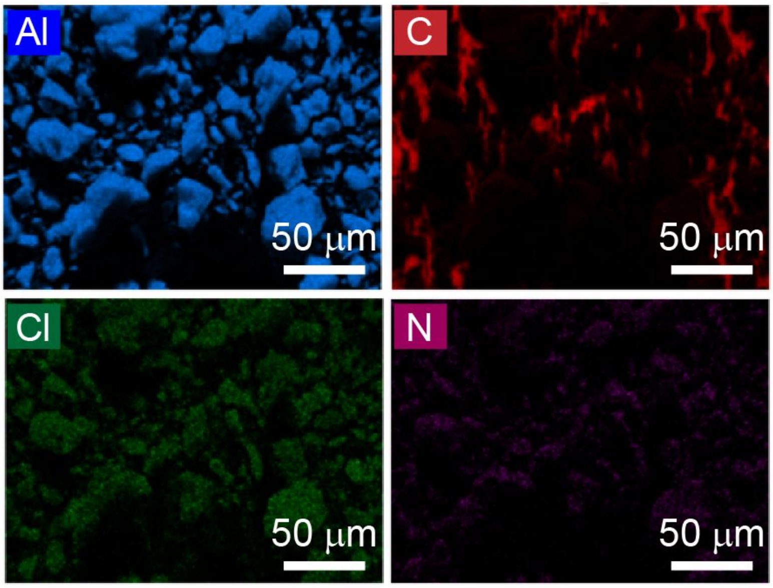

Figure S4. SEM-EDS chemical mapping of NHC@SILP-PMImAl$_2$O$_3$.

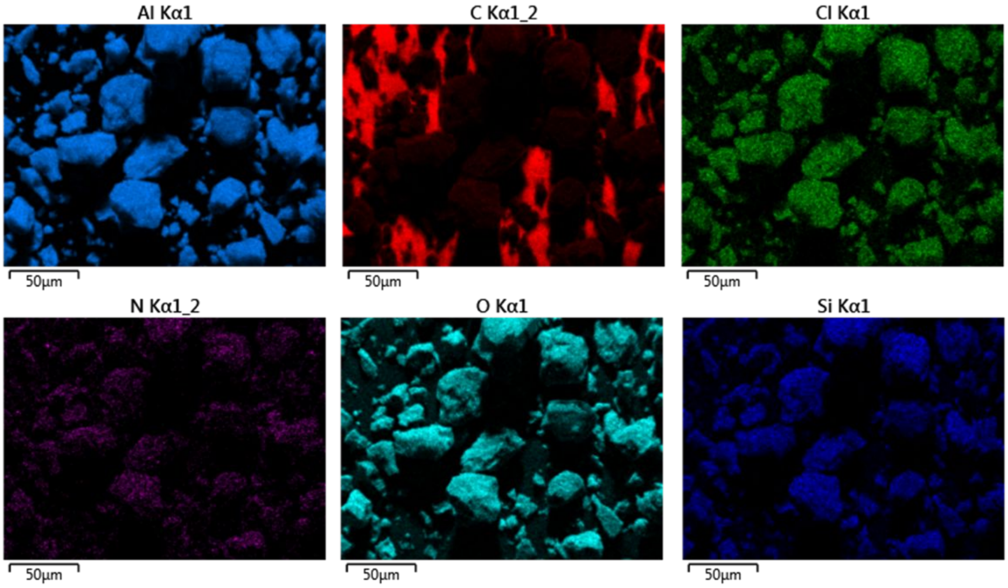

Figure S5. SEM and Chemical mapping of SILP-PPy-Al_2O_3 catalyst

5. BET analysis of SILP-Al_2O_3

The specific surface area was measured and compared with the one without organic linker, BET analysis confirms that the ionic liquid was successfully incorporated in Al_2O_3 once there has been a decrease in the pore volume and specific surface area as indicated in Table S1, caused by blockage or filling of the pores when compared with the unmodified Al_2O_3.

Table S1. BET Surface area, pore size and pore volume BJH adsorption of catalysts

Catalyst	Surface area (m^2/g)	Pore size (Â)	Pore volume(cm^3/g)
Al_2O_3	109.7	69.7	0.25
NHC@SILP-PMImAl$_2$O$_3$	16.3	70.2	0.05
SILP-PPyAl$_2$O$_3$	15.2	82.8	0.06

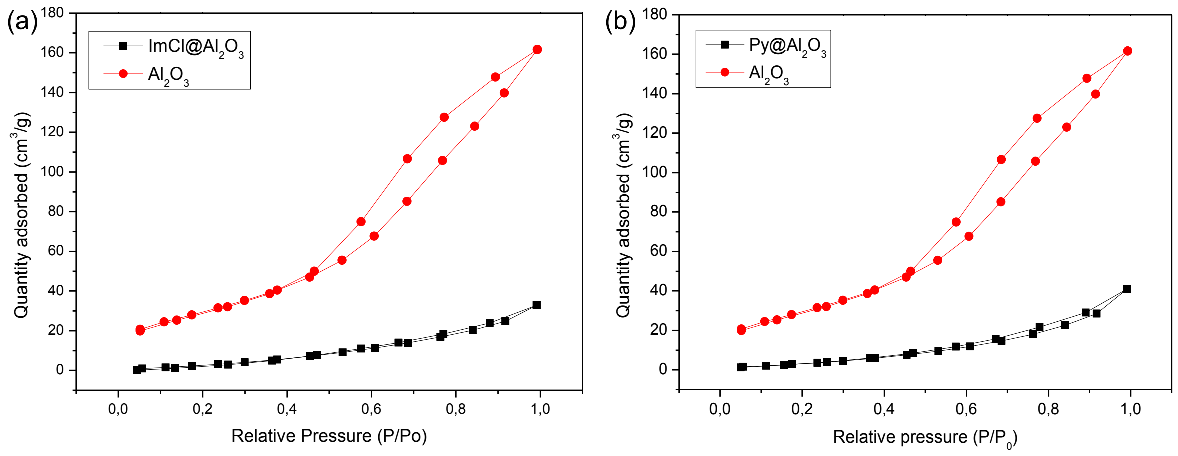

Figure S6. FTIR spectra of (a) NHC@SILP-PMImAl$_2$O$_3$ and (b) SILP-PPyAl$_2$O$_3$.

6. DFT calculations

A comprehensive computational study employing Density Functional Theory (DFT) was conducted using Gaussian 16, to investigate the interaction between aluminum oxide and epichlorohydrin in an ionic liquid environment. The structure of bulk γ-Al$_2$O$_3$ (001) was constructed based on the model described by Acikgoz and colleagues.[2] Calculations utilized the B3LYP functional and the 6-31G basis set, with an additional single-point calculation at the B3LYP/DEF2SVP level to ensure precision and reliability. Molecular species were optimized under stringent convergence criteria, accounting for zero-point energy (ZPE) contributions. Energies were calculated relative to the sum of isolated reactants, establishing the zero-energy point. Critical points were validated through vibrational analysis.

7- Solid state ^{13}C-MAS NMR spectra of coated carbon-13 enriched BMIm.Cl on Al_2O_3.

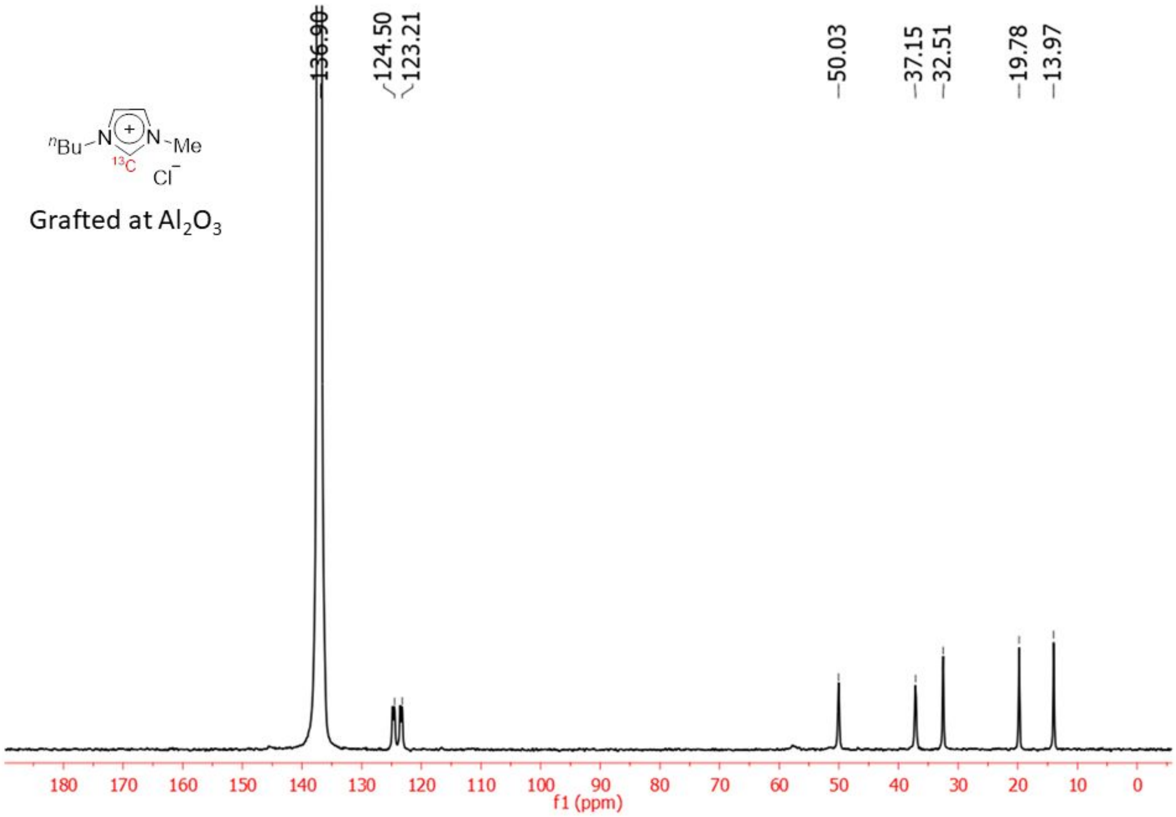

Figure S7. Solid state ^{13}C-MAS NMR spectra of grated carbon-13 enriched BMIm.Cl on Al_2O_3. The sample was prepared by missing carbon-13 enriched BMIm.Cl IL (140 mg) and Al_2O_3 (500 mg) in $CHCl_3$ (10 mL) for 20 h at RT. The obtained solid was filtered and dried under vacuum.

8- 2D ^{1}H-^{13}C HETCOR solid state NMR spectra of NHC@SILP-PMImAl$_2$O$_3$

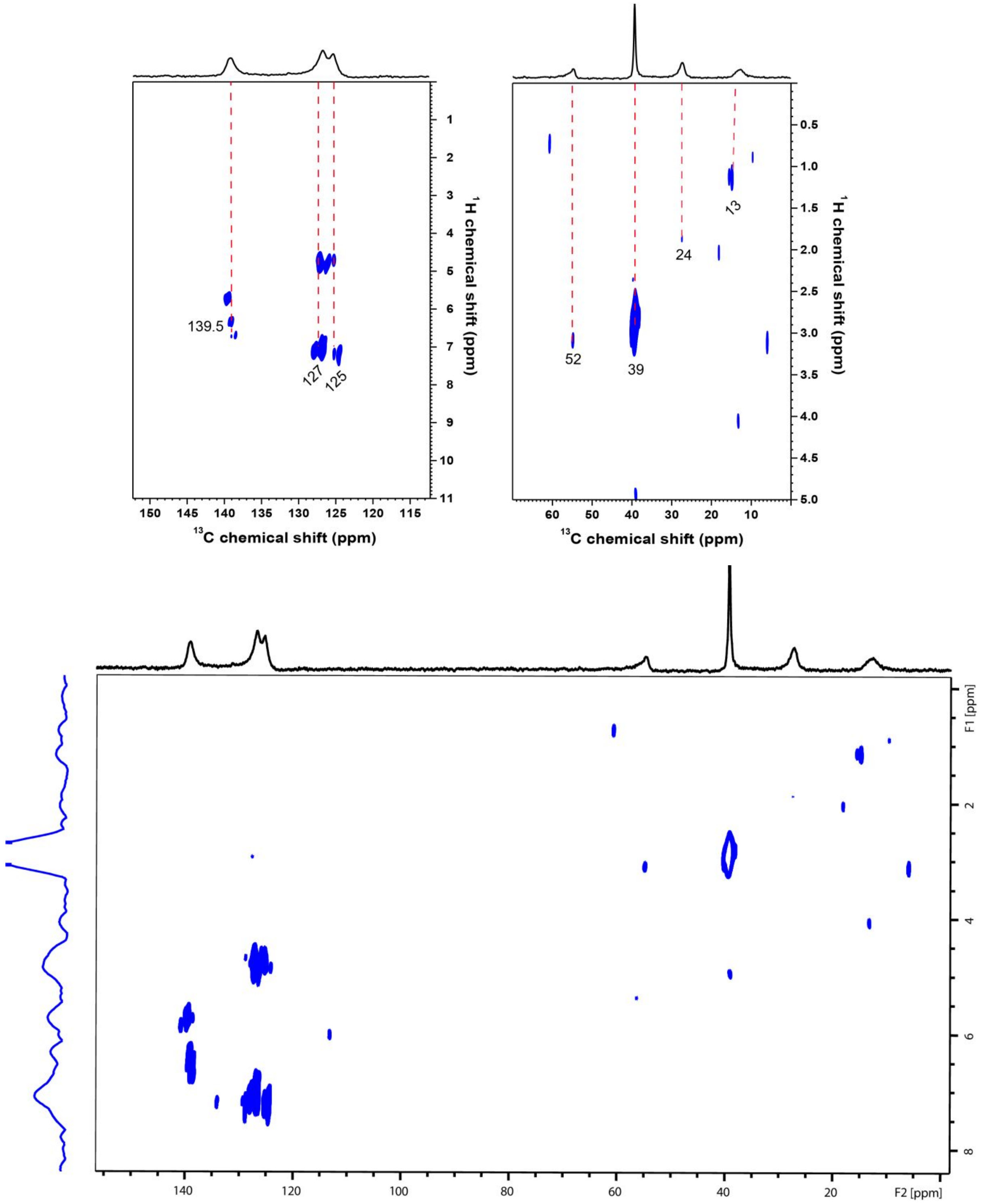

Figure S8. 2D ^{1}H-^{13}C HETCOR solid state NMR spectra of NHC@SILP-PMImAl$_2$O$_3$.

9. Surface composition of SILPs by AR-XPS analysis

The surface chemistry of the as-prepared SILPs was investigated using XPS. The procedures applied for data acquisition, calibration, correction, and analysis are available in the ESI, along with complementary results. It is important to emphasize that the stability of surface species under X-ray exposure was tested. Samples were subjected to 5 hours of exposure to a conventional X-ray source (Al-Kα at 250W), with no significant changes observed in the survey scans or any core-level spectra of either system. This indicates that both surfaces are resistant to beam damage, as well as the effects of emitted electrons from the filament. Additionally, beam damage was tested using a high flux of 1486.6 eV photons at the IPE-BEAMLINE/Sirius, further confirming the stability of the surface IL layers and the robustness of the IL-substrate interactions in both systems.

The depth distribution of elements in NHC@SILP-PMImAl2O3 and SILP-PPyAl2O3 were evaluated by AR-XPS data collected at three detection angles (10°, 45°, and 80° measured from surface normal) to extract bulk-sensitive (at 10°, with information depth > 7nm) and surface-sensitive (80° with information depth < 2nm) information. For each angle, the corrected areas of C 1s, N 1s, O 1s, Cl 2p, Al 2p and Si 2s regions were used to extract the relative concentrations shown in Figure S1 and the elemental ratios (Figure S2), whose comparative analysis enlightened the IL-support and cation-anion interactions at SILPs surfaces.

The AR-XPS data confirm the successful anchoring of both ILs onto the alumina surface, with consistent N, Cl, and Si concentrations detected across all angles. It is noteworthy that both O and Al amounts decrease from bulk to surface, while the IL-related elements become more abundant at shallow probing depths. Additionally, the aligned variations of O and Al and the opposite depth dependence of O and C indicate that most of the detected O is from alumina and that a representative part of the C 1s arises from IL layer with relatively low amounts of adventitious carbon on the fresh samples´ surface. Despite analyzed along with entire data set, O 1s and C 1s regions contributions were not used to support our results on depth-distribution of species to avoid misinterpretations. Additionally, the differences between samples were enhanced by comparing elemental ratios and their relative variations (Figure 4a)

The N/Al ratio (Figure S2) reflects the surface coverage and IL-support interaction. NHC@SILP-PMImAl$_2$O$_3$ exhibits a consistently higher N/Al ratio compared to SILP-PPyAl$_2$O$_3$

across all detection angles, in part due to the two N atoms present in the imidazolium ring compared to the single one in the pyridinium. The depth-dependences of N/Al ratio for each sample are compared in the bottom panel of Figure 4a by the relative variation with respect to the respective N/Al ratio values at 0°. For NHC@SILP-PMImAl_2O_3, the gradual increase in N/Al ratio with increasing detection angle suggests a uniform IL distribution, indicating efficient and homogeneous surface coverage due to strong interaction between the imidazolium cation and surface. This result is consistent with DFT calculations, which show the imidazolium cation in a tilted position.

In contrast, SILP-PPyAl_2O_3 shows a steeper increase in N/Al ratio for higher detection angle, along with the overall lower amount of IL-related species suggests weaker interaction between the pyridinium-based IL and the alumina support, leading to a less stable surface layer. The preferential orientation of the pyridinium-based IL may involve a tilted or upright configuration, minimizing contact with the support. This is consistent with DFT calculations, which show the pyridinium cation in a slightly upright geometry, positioned further away from the surface compared to the imidazolium cation

The N/Cl ratio (Figure S8) and its variation with respect to the nominal value (Figure 4a, top) reflects the cation-anion interactions. For SILP-PMImAl_2O_3, the minimal deviation of the N/Cl ratio from the nominal value of 2 indicates strong cation-anion interactions and short distances between them. On the other hand, SILP-PPyAl_2O_3 shows an increased N/Cl ratio at higher detection angles, indicating a greater cation-anion spatial separation and, consequently, weaker electrostatic interactions between the pyridinium cation and chloride anion. Moreover, all elemental ratios in the ESI point to a representative depletion of Cl across the surface of SILP-PPyAl_2O_3. This trend is consistent with the less stable and less uniform IL layer observed in SILP-PPyAl_2O_3. This further supports the conclusion that cation-anion interactions are weaker in the pyridinium-based system compared to the imidazolium one.

The N 1s spectra reinforce these findings. Both samples display a main peak corresponding to nitrogen within the imidazolium and pyridinium rings. However, in SILP-PMImAl_2O_3, a shoulder at lower BE suggests the formation of NHC species, indicating stronger covalent-like interactions between nitrogen and the alumina surface. To rule out the possible presence of neutral nitrogen species, which might be formed during X-ray exposure, stability tests were performed as discussed previously. In addition, the N 1s XPS spectra

were collected using two different excitation energies (1486 eV and 486 eV) to tune the surface sensitivity by selecting the kinetic energies of the photoelectrons emitted from the N core level (Figure S10). These tests revealed absence of neutral nitrogen species in both systems, even after prolonged X-ray exposure and provided insights into SILPs properties. For SILP-PMImAl_2O_3, it was observed an increase in the low-energy component for the most surface-sensitive data. This result confirms the relevant amount of nitrogen in this chemical environment at the outermost region of the imidazolium-modified surface, consistent with NHC formation and strong nitrogen-substrate interactions.

In contrast, SILP-PPyAl_2O_3 does not show significant changes between the two excitation energies, indicating that nitrogen is less concentrated at the surface and suggesting weaker nitrogen-substrate interactions. This finding, combined with the absence of a low binding energy shoulder in the N 1s spectra, supports the conclusion that the pyridinium IL forms weaker, less effective interactions with the alumina substrate.

The changes experienced by Al species due to surface modification were assessed by XPS data fitting and by XANES measurements at Al K-edge. The results corroborate the surface modification by IL and formation of multiple and active sites at SILPs surface, especially for SILP-PMImAl_2O_3.

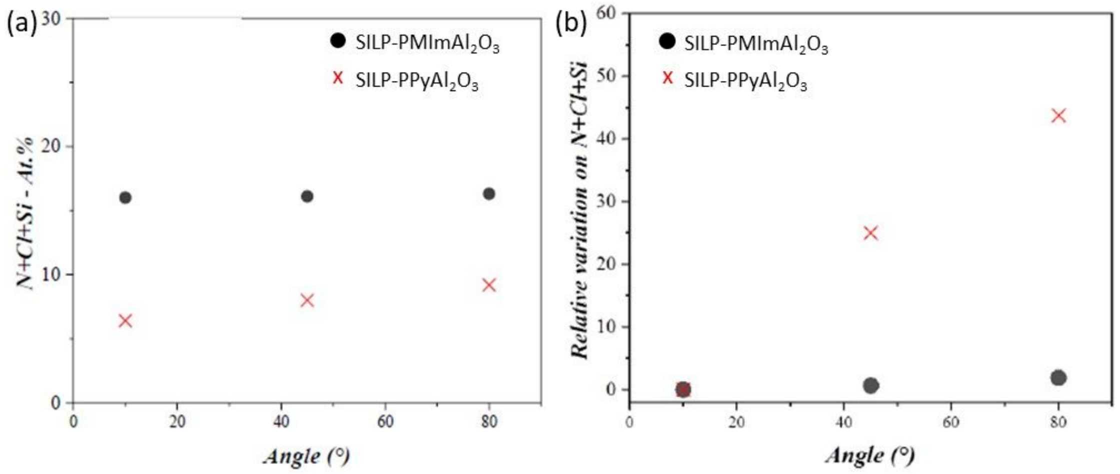

Figure S9: Surface chemical composition of N,Cl and Si of SILPs determined by Synchrotron AR-XPS analysis

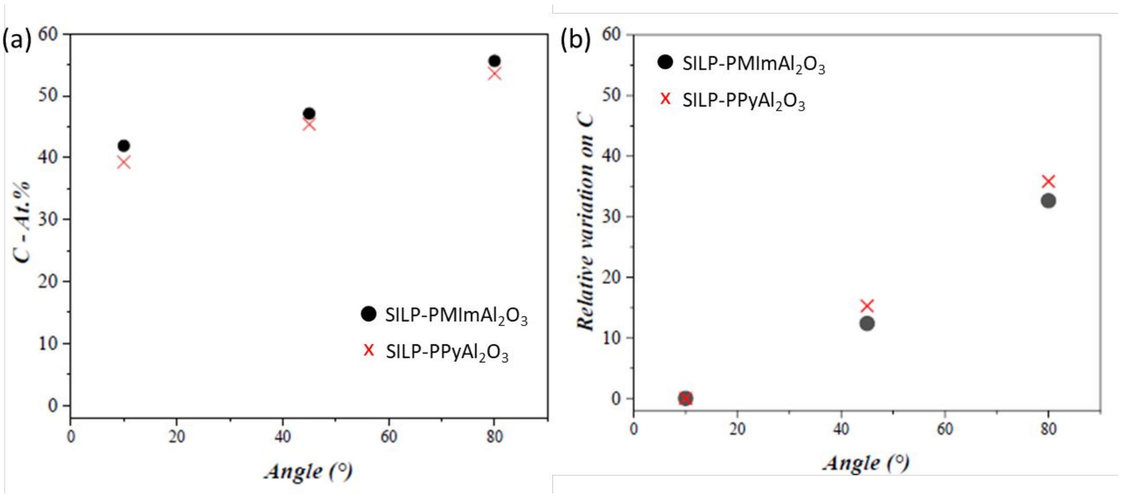

Figure S10: Surface chemical composition of C of SILPs determined by Synchrotron AR-XPS analysis

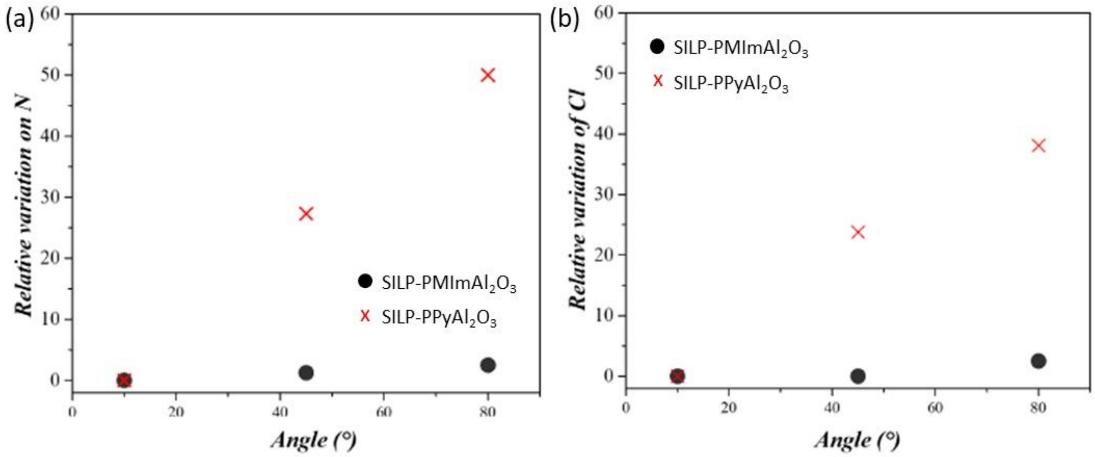

Figure S11: Surface chemical composition of N and Cl of SILPs determined by Synchrotron AR-XPS analysis

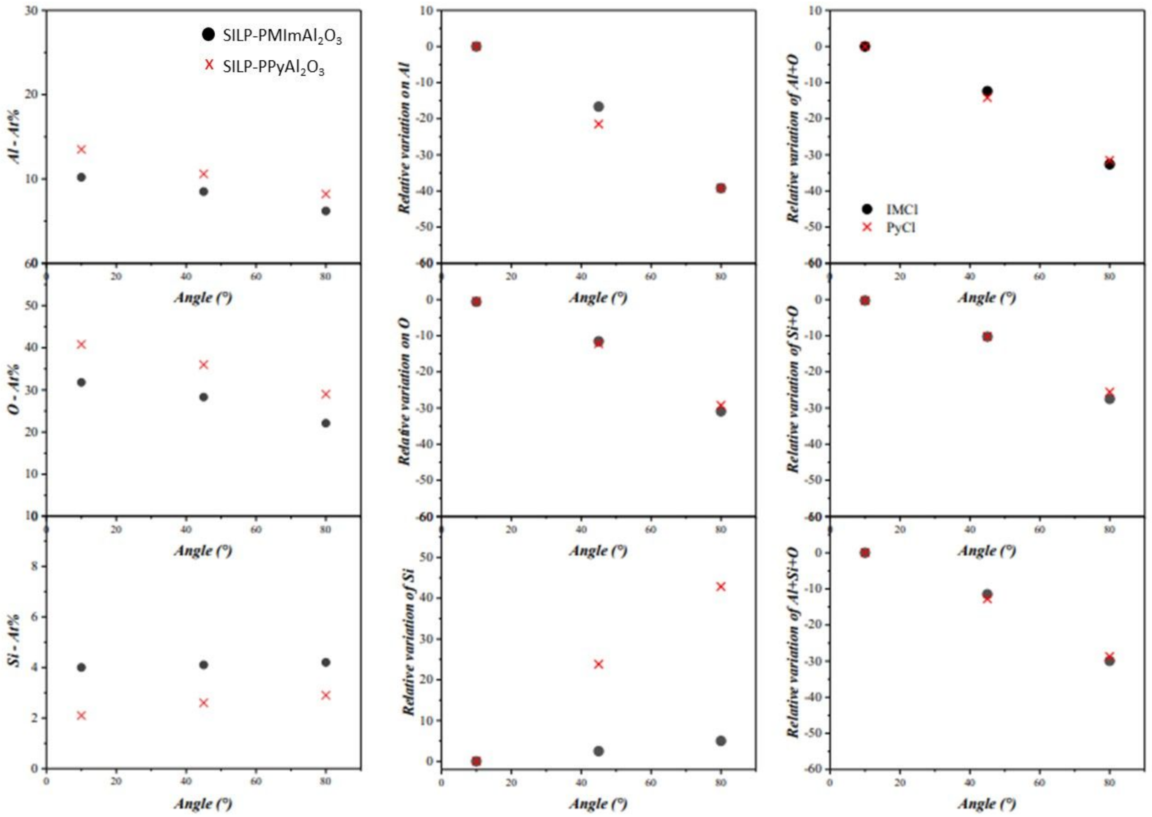

Figure 12: Surface chemical composition of Al, O and Si of SILPs determined by Synchrotron AR-XPS analysis.

10. FTIR analysis of SILPs

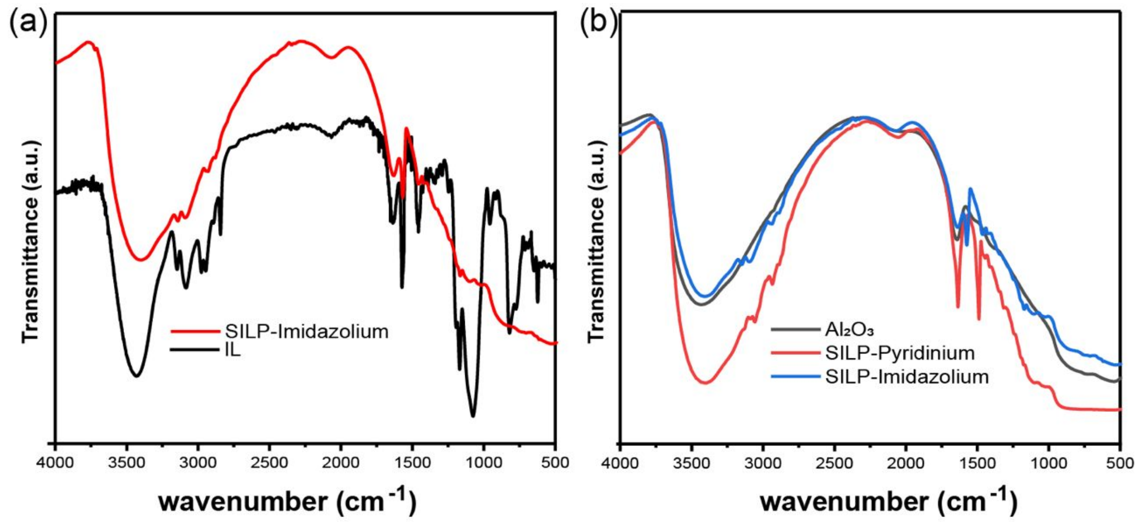

Figure S13. FTIR spectra of SILPs; (a) SILP-Imidazolium (NHC@SILP-PMImAl$_2$O$_3$) and IL(1-methyl-3-(3-(trimethoxysilyl)propyl)-imidazolium chloride), (b) SILP-Imidazolium (NHC@SILP-PMImAl$_2$O$_3$) and SILP-Pyridinium (SILP-PPyAl$_2$O$_3$)

11. DFT calculations for Adsorption energies of coordination modes of NHCs

Calculations within density functional theory (DFT) framework [1] were performed as implemented at the Vienna Ab initio Simulation Package (VASP 5.4.1)[3] [2-5] which treats the interactions between ions and electrons by the projector augmented wave method (PAW) [6,7].[4] The generalized gradient approximation (GGA) with the Perdew–Burke–Ernzerhof (PBE) exchange-correlation functional [8,9] was used in this study with a kinetic cut-off energy of 500 eV.[5] The Brillouin zone for such molecular system was sampled considered only the Γ k-point. The system under investigation was placed at the center of a 25 Å lattice cubic box with a minimum vacuum space between two neighboring systems of more than 12 Å to avoid possible interactions between periodic images. The atoms in the bottom layer were kept fixed at their bulk positions and all other atoms were allowed to relax without constraints and the geometry optimization was stopped when the electronic energy tolerance (10^{-6} eV) was reached, and the residual forces on each atom were less than 0.01 eV/Å. The van der Waals interactions (vdW) were included by using the semi-empirical D2 method [10].[6] The adsorption energy was calculated using the expression:

$$E(ads) = E(Al_2O_3 + C_7N_2H_{12}) - [E(Al_2O_3) + E(C_7N_2H_{12})],$$

where $E(Al_2O_3 + C_7N_2H_{12})$ is the total energy of the optimized system, $E(Al_2O_3)$ is the total energy of the Al_2O_3 cluster and $E(C_7N_2H_{12})$ is the total energy of the isolated $C_7N_2H_{12}$ molecule. Thus, a negative E(ads) value means an exothermic adsorption of the molecule on the Al_2O_3 cluster.

12. References

[1] L. Foppa, L. Luza, A. Gual, D. E. Weibel, D. Eberhardt, S. R. Teixeira, J. Dupont, *Dalton Trans.* **2015**, *44*, 2827-2834.

[2] M. Acikgoz, J. Harrell, M. Pavanello, *J. Phys. Chem. C* **2018**, *122*, 25314-25330.

[3] a) G. Kresse, J. Hafner, *Phys. Rev. B* **1993**, *47*, 558-561; b) G. Kresse, J. Hafner, *Phys. Rev. B* **1994**, *49*, 14251-14269.

[4] a) G. Kresse, D. Joubert, *Phys. Rev. B* **1999**, *59*, 1758-1775; b) J. P. Perdew, J. A. Chevary, S. H. Vosko, K. A. Jackson, M. R. Pederson, D. J. Singh, C. Fiolhais, *Phys. Rev. B* **1992**, *46*, 6671-6687.

[5] a) A. D. Becke, *Phys. Rev. A* **1988**, *38*, 3098-3100; b) J. P. Perdew, K. Burke, M. Ernzerhof, *Physical Review Letters* **1996**, *77*, 3865-3868.

[6] S. Grimme, *J. Comput. Chem.* **2006**, *27*, 1787-1799.
